# Supplementary material for: Whole-genome sequencing and phylogenetic analysis of Salmonella isolated from pullets through final raw product in the processing plant of a conventional broiler complex: a longitudinal study
Source: Microbiol Spectr. 2025 Jan 14;13(2):e02090-24. doi: 10.1128/spectrum.02090-24 (PMC11792521; doi:10.1128/spectrum.02090-24)
Supplement: Supplemental figure and tables — Fig. S1 (A-K); Tables S1 and S2. [file spectrum.02090-24-s0001.docx]

**SUPPLEMENTARY MATERIALS**


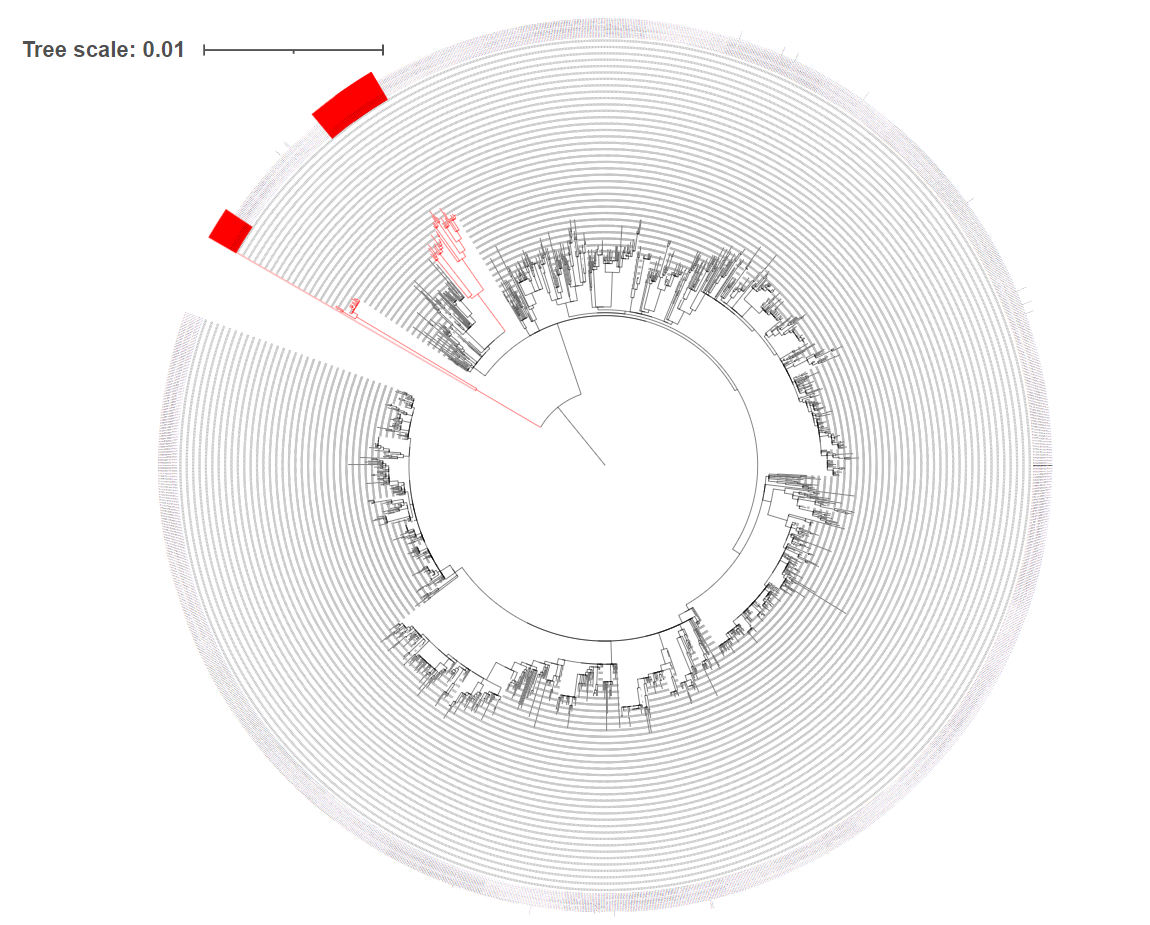


**(A)** **Phylogenetic tree of 64 *Salmonella* Kentucky isolates from this study and 1651 NCBI genomes.**


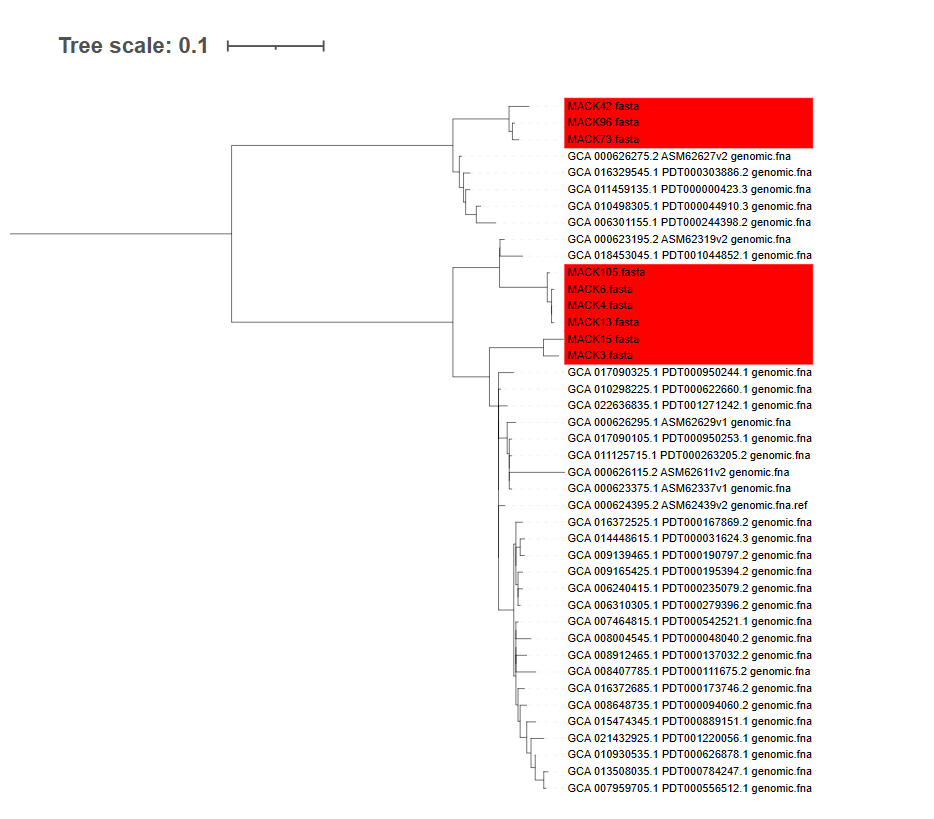


**(B) Phylogenetic tree of 9 *Salmonella* Enteritidis isolates from this study and 33 selected NCBI genomes.**

**
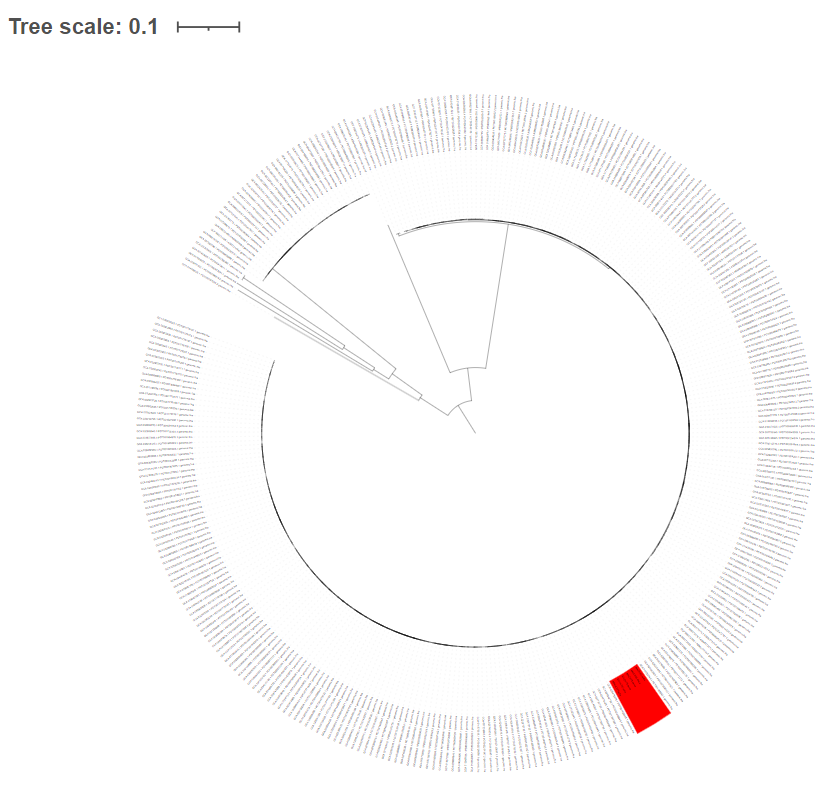
**

**(C) Phylogenetic tree of 6 *Salmonella* Alachua isolates from this study and all 314 selected NCBI genomes.**


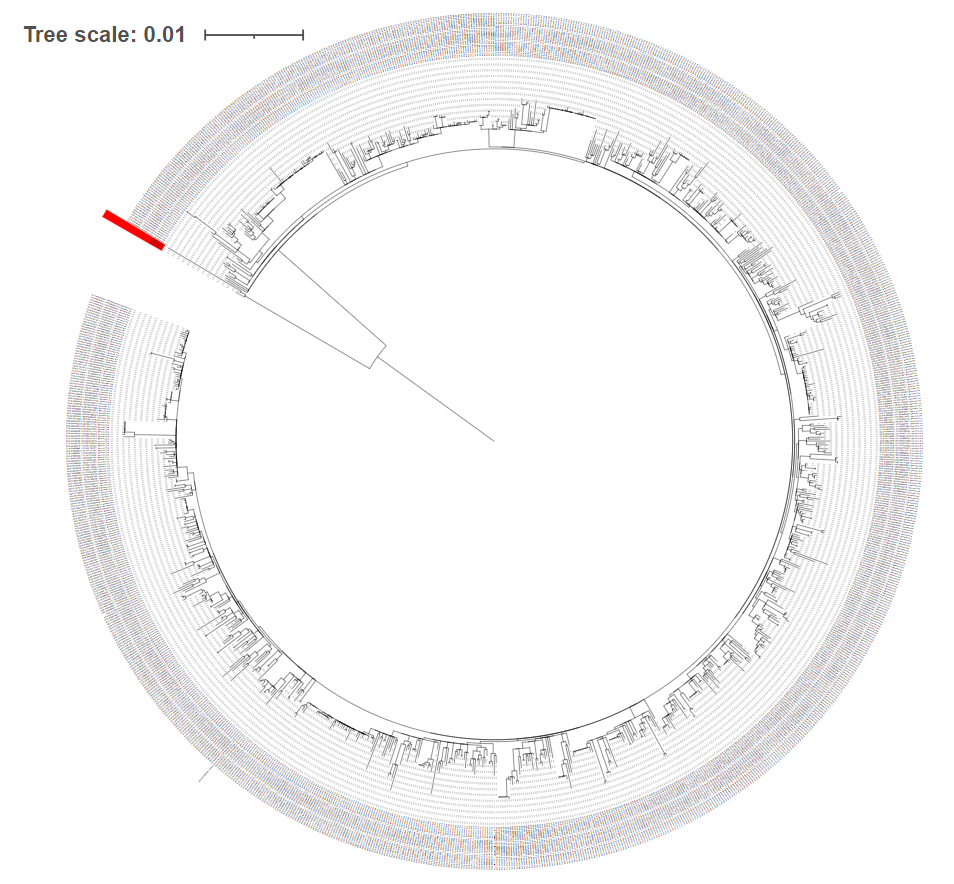


**(D) Phylogenetic tree of 3 *Salmonella* Typhimurium isolates from this study and selected 1032 NCBI genomes.**


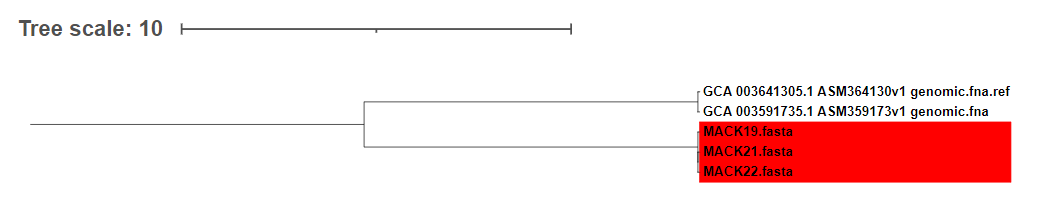


**(E) Phylogenetic tree of *a Salmonella* Mbandaka isolates from this study and 2 selected NCBI genomes.**


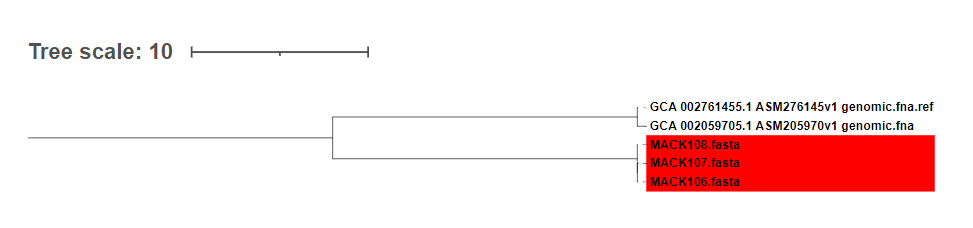


**(F) Phylogenetic tree of 3 *Salmonella* Montevideo isolates from this study and 2 selected NCBI genomes.**


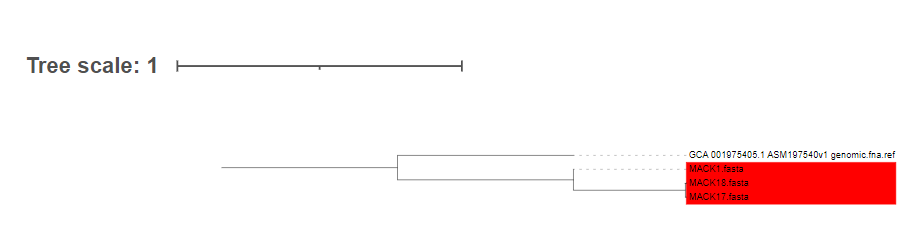


**(G) Phylogenetic tree of 3 *Salmonella* Johannesburg isolates from this study and selected NCBI genome.**


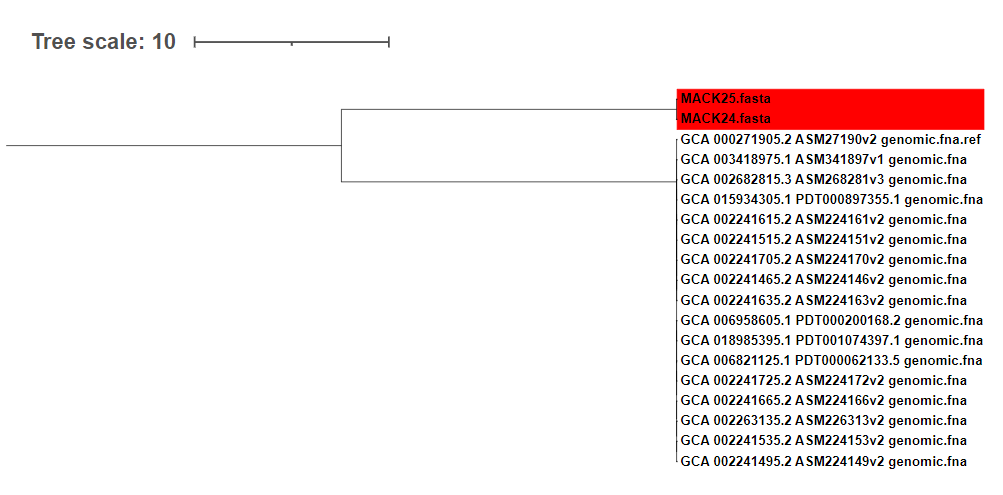


**(H) Phylogenetic tree of 2 *Salmonella* Newport isolates from this study and 17 NCBI genomes.**


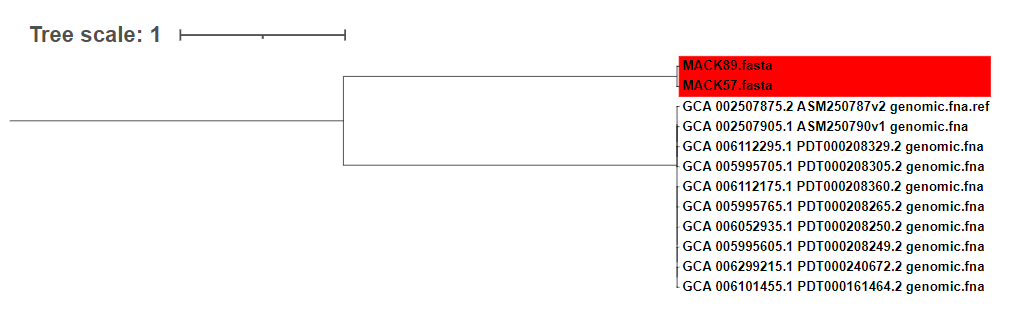


**(I) Phylogenetic tree of 2 *Salmonella* Uganda isolates from this study and 10 selected NCBI genomes.**


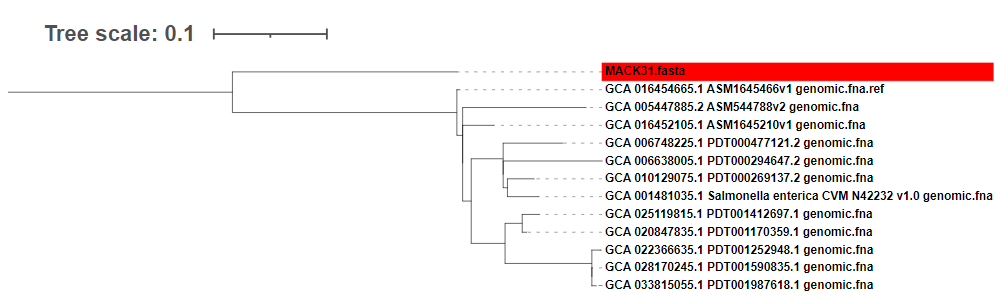


**(J) Phylogenetic tree of *a Salmonella* Senftenberg isolates from this study and 12 selected NCBI genomes.**


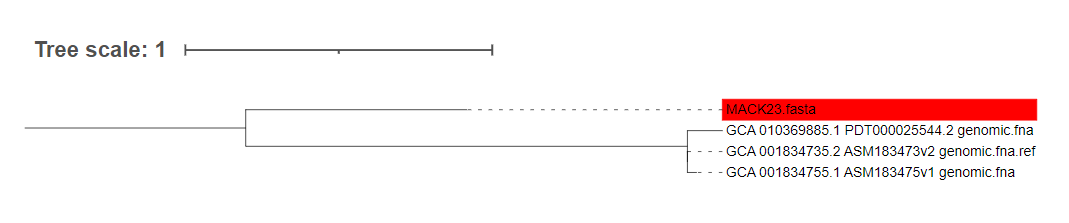


**(K) Phylogenetic tree of *a Salmonella* Inverness isolates from this study and 3 selected NCBI genomes.**

Figure 1: Phylogenetic tree of *Salmonella* isolates from this study (highlighted with red color) along with selected NCBI strains based on each serotype.

(A) *S.* Kentucky strains from this study with 1651 NCBI strains, (B) *S.* Enteritidis strains from this study with 33 NCBI strains, (C) *S.* Alachua strains from this study with 314 NCBI strains (D) *S*. Typhimurium strains from this study with 1032 NCBI strains, (E) *S.* Mbandaka strains from this study with 2 NCBI strains, (F) *S.* Montevideo strains from this study with 2 NCBI strains, (G) *S.* Newport strains from this study with 17 NCBI strains (H) *S.* Senftenberg strains from this study with 12 NCBI strains (I) *S.* Uganda strains from this study with 10 NCBI strains, (J) *S.* Ohio strains from this study with 2 NCBI strains, and (K) *S.* Inverness strains from this study with 3 NCBI strains.

**Table 1: List of *Salmonella* isolates from this study with serotypes, sample types and stages.**

| **S. No.** | **Sample no.** | **Serotypes** | **Sample types** | **Stages** |
| --- | --- | --- | --- | --- |
|  | 28 | Johannesburg | Incubator swab | hatchery |
|  | 31 | Ohio | Incubator swab | hatchery |
|  | 35 | Enteritidis | Halo swab near ember | hatchery |
|  | 37 | Enteritidis | Composite egg samples from egg remover (near embrex) | hatchery |
|  | 38 | Kentucky | Composite egg samples from egg remover (near embrex) | hatchery |
|  | 42 | Enteritidis | Clean egg buggy/trays without eggs | hatchery |
|  | 47 | Kentucky | Chick trays (dirty) delivered from broiler farms | hatchery |
|  | 48 | Kentucky | Chick trays (dirty) delivered from broiler farms | hatchery |
|  | 49 | Kentucky | Chick trays (dirty) delivered from broiler farms | hatchery |
|  | 93A | N/A (9:z29:-) | fly paper in hatcher 1 | hatchery |
|  | 93D | N/A (9:z29:-) | fly paper in hatcher 1 | hatchery |
|  | 95 | Kentucky | bootswab from setter 2 | hatchery |
|  | 98 | Enteritidis | bootswab from hatcher 3 | hatchery |
|  | 101 | Kentucky | bootswab from hallway/corridor | hatchery |
|  | 116A | Enteritidis | fly paper from dumpster | hatchery |
|  | 116D | Kentucky | fly paper from dumpster | hatchery |
|  | 123A | Johannesburg | mouse intestine | hatchery |
|  | 123C | Johannesburg | mouse intestine | hatchery |
|  | 29 | Mbandaka | feces outside | pullet farms |
|  | 106 | Kentucky | water puddles outside | pullet farms |
|  | 124 | Mbandaka | fan exhaust swab inside | pullet farms |
|  | 133 | Mbandaka | fan exhaust swab inside | pullet farms |
|  | 13 | Inverness | soil sample | breeder |
|  | 20 | Newport | rat feces | breeder |
|  | 22 | Newport | rat feces | breeder |
|  | 28 | Kentucky | drainage water | breeder |
|  | 110 | Kentucky | fan exhaust swab inside | breeder |
|  | 115 | Kentucky | boot swab | breeder |
|  | 129 | Kentucky | litter | breeder |
|  | 5A | Kentucky | beetle trap (only litter) | breeder |
|  | 5C | Senftenberg or Dessau (O-1, 3, 19 H1 - g, st H2-) | beetle trap (only litter) | breeder |
|  | 18 | Kentucky | fly paper inside | breeder |
|  | 23 | Kentucky | beetle trap (only litter) | breeder |
|  | 47 | Kentucky | swab from nest box | breeder |
|  | 51 | Kentucky | swab from egg collection conveyer belt | breeder |
|  | 59 | Kentucky | bootswab | breeder |
|  | 64 | Kentucky | fan exhaust swab inside | breeder |
|  | 78 | Kentucky | boot swab | breeder |
|  | 81 | Kentucky | swab from nest box | breeder |
|  | 86 | Kentucky | dirty egg wipe paper | breeder |
|  | 93 | Kentucky | fly paper outside | breeder |
|  | 114 | Enteritidis | water drainage | breeder |
|  | 128 | Typhimurium | soil sample | breeder |
|  | 138 | Typhimurium | water drainage | breeder |
|  | 143 | Typhimurium | water puddles | breeder |
|  | 17 | Kentucky | swab from egg collection conveyer belt | breeder |
|  | 101 | Kentucky | crease in curtain, wildbird/rodent feces | breeder |
|  | 102 | Kentucky | wildbird feces | breeder |
|  | 14 | Enteritidis | ants | broiler farms |
|  | 1109 | Montevideo | drainage water | broiler farms |
|  | 1110 | Montevideo | drainage water | broiler farms |
|  | 1111 | Montevideo | feces, deer | broiler farms |
|  | 253 | Kentucky | fan exhaust swab inside | broiler farms |
|  | 254 | Kentucky | fan exhaust swab inside | broiler farms |
|  | 520 | Enteritidis | fan exhaust swab inside | broiler farms |
|  | 54 | Kentucky | carcass rinses (pot-picking) | processing |
|  | 55 | Kentucky | carcass rinses (pot-picking) | processing |
|  | 59 | Kentucky | carcass rinses (pot-picking) | processing |
|  | 61 |  | carcass rinses (pot-picking) | processing |
|  | 62 |  | carcass rinses (pot-picking) | processing |
|  | 63 |  | carcass rinses (pot-picking) | processing |
|  | 64 |  | carcass rinses (pot-picking) | processing |
|  | 65 |  | carcass rinses (pot-picking) | processing |
|  | 66 |  | carcass rinses (pot-picking) | processing |
|  | 67 |  | carcass rinses (pot-picking) | processing |
|  | 68 |  | carcass rinses (pot-picking) | processing |
|  | 69 |  | carcass rinses (pot-picking) | processing |
|  | 70 |  | carcass rinses (pot-picking) | processing |
|  | 71 |  | carcass rinses (pot-picking) | processing |
|  | 73 |  | carcass rinses (pot-picking) | processing |
|  | 75 |  | carcass rinses (pot-picking) | processing |
|  | 76 |  | carcass rinses (pot-picking) | processing |
|  | 77 |  | carcass rinses (pot-picking) | processing |
|  | 98 |  | carcass rinses (pot-chilling) | processing |
|  | 106 | Kentucky | carcass rinses (pot-chilling) | processing |
|  | 107 | Kentucky | carcass rinses (pot-chilling) | processing |
|  | 207 | Kentucky | carcass rinses (pot-chilling) | processing |
|  | 114 |  | moving belt swab (live birds hang area) | processing |
|  | 115 | Kentucky | moving belt swab (live birds hang area) | processing |
|  | 116 | Kentucky | moving belt swab (live birds hang area) | processing |
|  | 119 | Alachua | moving belt swab (live birds hang area) | processing |
|  | 120 | Kentucky | fresh feces from live birds hang area | processing |
|  | 129 |  | swab from moving belt (loading dock area) | processing |
|  | 130 | Kentucky | swab from moving belt (loading dock area) | processing |
|  | 131 | Kentucky | swab from moving belt (loading dock area) | processing |
|  | 132 | Kentucky | swab from moving belt (loading dock area) | processing |
|  | 133 |  | swab from moving belt (loading dock area) | processing |
|  | 134 |  | swab from moving belt (loading dock area) | processing |
|  | 135 |  | swab from moving belt (loading dock area) | processing |
|  | 136 |  | swab from moving belt (loading dock area) | processing |
|  | 137 |  | swab from moving belt (loading dock area) | processing |
|  | 138 |  | swab from moving belt (loading dock area) | processing |
|  | 139 |  | swab from moving belt (loading dock area) | processing |
|  | 140 |  | swab from moving belt (loading dock area) | processing |
|  | 141 |  | swab from moving belt (loading dock area) | processing |
|  | 142 |  | swab from moving belt (loading dock area) | processing |
|  | 143 |  | swab from moving belt (loading dock area) | processing |
|  | 144 |  | swab from moving belt (loading dock area) | processing |
|  | 149 | Alachua | transport cage swab just before loading birds | transport |
|  | 155 |  | transport cage while in trucks | transport |
|  | 160 | Kentucky | transport cage while in trucks | transport |
|  | 163 |  | transport cage while in trucks | transport |
|  | 165 | Kentucky | transport cage while in trucks | transport |
|  | 166 | Kentucky | transport cage while in trucks | transport |
|  | 167 |  | transport cage while in trucks | transport |
|  | 168 |  | transport cage while in trucks | transport |
|  | 220 | Kentucky | carcass rinses (post-picking) | processing |
|  | 221 | Kentucky | carcass rinses (post-picking) | processing |
|  | 222 | Kentucky | carcass rinses (post-picking) | processing |
|  | 223 |  | carcass rinses (post-picking) | processing |
|  | 224 |  | carcass rinses (post-picking) | processing |
|  | 225 |  | carcass rinses (post-picking) | processing |
|  | 226 |  | carcass rinses (post-picking) | processing |
|  | 227 |  | carcass rinses (post-picking) | processing |
|  | 228 |  | carcass rinses (post-picking) | processing |
|  | 229 |  | carcass rinses (post-picking) | processing |
|  | 230 |  | carcass rinses (post-picking) | processing |
|  | 231 |  | carcass rinses (post-picking) | processing |
|  | 232 |  | carcass rinses (post-picking) | processing |
|  | 234 |  | carcass rinses (post-picking) | processing |
|  | 235 |  | carcass rinses (post-picking) | processing |
|  | 236 |  | carcass rinses (post-picking) | processing |
|  | 237A |  | carcass rinses (post-picking) | processing |
|  | 237C |  | carcass rinses (post-picking) | processing |
|  | 238 |  | carcass rinses (post-picking) | processing |
|  | 276 | Kentucky | carcass rinses (post-chilling) | processing |
|  | 283 | Kentucky | carcass rinses (post-chilling) | processing |
|  | 329 |  | moving belt swab (live birds hang area) | processing |
|  | 330 | Kentucky | moving belt swab (live birds hang area) | processing |
|  | 331 | Enteritidis | moving belt swab (live birds hang area) | processing |
|  | 332 |  | moving belt swab (live birds hang area) | processing |
|  | 333 |  | moving belt swab (live birds hang area) | processing |
|  | 334 |  | moving belt swab (live birds hang area) | processing |
|  | 335 | Kentucky | moving belt swab (live birds hang area) | processing |
|  | 336 |  | moving belt swab (live birds hang area) | processing |
|  | 337 |  | moving belt swab (live birds hang area) | processing |
|  | 338 |  | moving belt swab (live birds hang area) | processing |
|  | 340 | Alachua | fresh feces from live birds hang area | processing |
|  | 350 | Alachua | swab from floor of transport cages | transport |
|  | 354A | Alachua | swab from floor of transport cages | transport |
|  | 354C | Alachua | swab from floor of transport cages | transport |
|  | 356 | Kentucky | swab from floor of transport cages | transport |
|  | 3 |  | swab from cage floor on truck | transport |
|  | 4 | Kentucky | swab from cage floor on truck | transport |
|  | 5 |  | swab from cage floor on truck | transport |
|  | 6 |  | swab from cage floor on truck | transport |
|  | 7 | Kentucky | swab from cage floor on truck | transport |
|  | 8 |  | swab from cage floor on truck | transport |
|  | 9 | Kentucky | swab from cage floor on truck | transport |
|  | 10 |  | swab from cage floor on truck | transport |
|  | 11 |  | swab from cage floor on truck | transport |
|  | 12 |  | swab from cage floor on truck | transport |
|  | 13 |  | swab from cage floor on truck | transport |
|  | 14 |  | swab from cage floor on truck | transport |
|  | 15 |  | swab from cage floor on truck | transport |
|  | 16 |  | swab from cage floor on truck | transport |
|  | 17 |  | swab from cage floor on truck | transport |
|  | 18 |  | swab from cage floor on truck | transport |
|  | 19 |  | swab from cage floor on truck | transport |
|  | 20 |  | swab from cage floor on truck | transport |
|  | 21 |  | swab from cage floor on truck | transport |
|  | 22 |  | swab from cage floor on truck | transport |
|  | 23 |  | swab from cage floor on truck | transport |
|  | 24 |  | swab from cage floor on truck | transport |
|  | 25 |  | swab from cage floor on truck | transport |
|  | 29 | Kentucky | fecal sample from truck | transport |
|  | 30 |  | fecal sample from truck | transport |
|  | 31 | Kentucky | fecal sample from truck | transport |
|  | 35 |  | fecal sample from truck | transport |
|  | 37 | Kentucky | fecal sample from truck | transport |
|  | 38 |  | fecal sample from truck | transport |
|  | 56 | Kentucky | fecal sample from live birds hang area | processing |
|  | 62 | Kentucky | moving belt swab (live birds hang area) | processing |
|  | 63 | Kentucky | moving belt swab (live birds hang area) | processing |
|  | 64 | Uganda | moving belt swab (live birds hang area) | processing |
|  | 65 |  | moving belt swab (live birds hang area) | processing |
|  | 66 |  | moving belt swab (live birds hang area) | processing |
|  | 67 |  | moving belt swab (live birds hang area) | processing |
|  | 68 |  | moving belt swab (live birds hang area) | processing |
|  | 69 |  | moving belt swab (live birds hang area) | processing |
|  | 75 | Kentucky | carcass rinses (post-picking) | processing |
|  | 76 |  | carcass rinses (post-picking) | processing |
|  | 77 |  | carcass rinses (post-picking) | processing |
|  | 78 |  | carcass rinses (post-picking) | processing |
|  | 79 | Kentucky | carcass rinses (post-picking) | processing |
|  | 80 | Kentucky | carcass rinses (post-picking) | processing |
|  | 81 |  | carcass rinses (post-picking) | processing |
|  | 82 |  | carcass rinses (post-picking) | processing |
|  | 83 |  | carcass rinses (post-picking) | processing |
|  | 84 |  | carcass rinses (post-picking) | processing |
|  | 85 |  | carcass rinses (post-picking) | processing |
|  | 86 |  | carcass rinses (post-picking) | processing |
|  | 88 |  | carcass rinses (post-picking) | processing |
|  | 89 |  | carcass rinses (post-picking) | processing |
|  | 90 |  | carcass rinses (post-picking) | processing |
|  | 92 |  | carcass rinses (post-picking) | processing |
|  | 93 |  | carcass rinses (post-picking) | processing |

**Table 2: List of 100 representative *Salmonella* isolates that were whole genome sequenced with KmerF Inder results**

| **Sample** | **ID** | **Serotype** | **Sample types** | **Stages** | **Template** | **Query coverage** | **Template coverage** |
| --- | --- | --- | --- | --- | --- | --- | --- |
| MACK1 | 28 | Johannesburg | Incubator swab | hatchery | *Salmonella* enterica subsp. enterica serovar Johannesburg str. ST203 | 94.97 | 97.48 |
| MACK2 | 31 | Ohio | Incubator swab | hatchery | *Salmonella* enterica subsp. enterica strain SA20120345 | 94.75 | 98.65 |
| MACK3 | 35 | Enteritidis | Halo swab near ember | hatchery | *Salmonella* enterica subsp. enterica serovar Enteritidis str. EC20120002 | 98.03 | 99.96 |
| MACK4 | 37 | Enteritidis | Composite egg samples from egg remover (near embrex) | hatchery | *Salmonella* enterica subsp. enterica serovar Enteritidis str. EC20120005 | 97.03 | 99.99 |
| MACK5 | 38 | Kentucky | Composite egg samples from egg remover (near embrex) | hatchery | *Salmonella* enterica subsp. enterica serovar Kentucky strain CVM 30177 | 93.38 | 99.93 |
| MACK6 | 42 | Enteritidis | Clean egg buggy/trays without eggs | hatchery | *Salmonella* enterica subsp. enterica serovar Enteritidis str. EC20120005 | 97.3 | 99.98 |
| MACK7 | 47 | Kentucky | Chick trays (dirty) delivered from broiler farms | hatchery | *Salmonella* enterica subsp. enterica serovar Kentucky strain CVM 30177 | 92.93 | 99.92 |
| MACK8 | 48 | Kentucky | Chick trays (dirty) delivered from broiler farms | hatchery | *Salmonella* enterica subsp. enterica serovar Kentucky strain CVM 30177 | 93.38 | 99.92 |
| MACK9 | 49 | Kentucky | Chick trays (dirty) delivered from broiler farms | hatchery | *Salmonella* enterica subsp. enterica serovar Kentucky strain CVM 30177 | 93.38 | 99.92 |
| MACK10 | 93A | N/A (9:z29:-) | fly paper in hatcher 1 | hatchery | *Salmonella* enterica strain SalSpp_sample_05_No.1 | 98.28 | 96.25 |
| MACK11 | 93D | N/A (9:z29:-) | fly paper in hatcher 1 | hatchery | *Salmonella* enterica strain SalSpp_sample_05_No.1 | 98.28 | 96.25 |
| MACK12 | 95 | Kentucky | bootswab from setter 2 | hatchery | *Salmonella* enterica subsp. enterica serovar Kentucky strain CVM 30177 | 93.37 | 99.92 |
| MACK13 | 98 | Enteritidis | bootswab from hatcher 3 | hatchery | *Salmonella* enterica subsp. enterica serovar Enteritidis str. EC20120005 | 97.29 | 99.99 |
| MACK14 | 101 | Kentucky | bootswab from hallway/corridor | hatchery | *Salmonella* enterica subsp. enterica serovar Kentucky strain CVM 30177 | 93.38 | 99.93 |
| MACK15 | 116A | Enteritidis | fly paper from dumpster | hatchery | *Salmonella* enterica subsp. enterica serovar Enteritidis str. EC20120002 | 98.79 | 99.88 |
| MACK16 | 116D | Kentucky | fly paper from dumpster | hatchery | *Salmonella* enterica subsp. enterica serovar Kentucky strain CVM 30177 | 93.37 | 99.92 |
| MACK17 | 123A | Johannesburg | mouse intestine | hatchery | *Salmonella* enterica subsp. enterica serovar Johannesburg str. ST203 | 95.63 | 97.18 |
| MACK18 | 123C | Johannesburg | mouse intestine | hatchery | *Salmonella* enterica subsp. enterica serovar Johannesburg str. ST203 | 95.67 | 97.18 |
| MACK19 | 29A | Mbandaka | feces outside | pullet farms | *Salmonella* enterica subsp. enterica strain 11TTU1615b | 97.95 | 93.99 |
| MACK20 | 106 | Kentucky | water puddles outside | pullet farms | *Salmonella* enterica subsp. enterica serovar Kentucky strain CVM 30177 | 95.67 | 99.92 |
| MACK21 | 124 | Mbandaka | fan exhaust swab inside | pullet farms | *Salmonella* enterica subsp. enterica strain 11TTU1615b | 95.75 | 94 |
| MACK22 | 133 | Mbandaka | fan exhaust swab inside | pullet farms | *Salmonella* enterica subsp. enterica strain 11TTU1615b | 97.94 | 93.51 |
| MACK23 | 13 | Inverness | soil sample | breeder farms | *Salmonella* enterica subsp. enterica serovar Inverness strain CFSAN044911 | 95.79 | 97.23 |
| MACK24 | 20 | Newport | rat feces | breeder farms | *Salmonella* enterica subsp. enterica serovar Newport str. Levine 15 | 95.65 | 97.68 |
| MACK25 | 22 | Newport | rat feces | breeder farms | *Salmonella* enterica subsp. enterica serovar Newport str. Levine 15 | 97.7 | 97.68 |
| MACK26 | 28 | Kentucky | drainage water | breeder farms | *Salmonella* enterica subsp. enterica serovar Kentucky strain CVM 30177 | 93.36 | 99.93 |
| MACK27 | 110 | Kentucky | fan exhaust swab inside | breeder farms | *Salmonella* enterica subsp. enterica serovar Kentucky strain CVM 30177 | 93.38 | 99.92 |
| MACK28 | 115 | Kentucky | boot swab | breeder farms | *Salmonella* enterica subsp. enterica serovar Kentucky strain CVM 30177 | 93.36 | 99.92 |
| MACK29 | 129 | Kentucky | litter | breeder farms | *Salmonella* enterica subsp. enterica serovar Kentucky strain CVM 30177 | 93.3 | 99.93 |
| MACK30 | 5A | Kentucky | beetle trap (only litter) | breeder farms | *Salmonella* enterica subsp. enterica serovar Kentucky strain CVM 30177 | 93.36 |  |
| MACK31 | 5C | Senftenberg or Dessau (O-1, 3, 19 H1 - g, st H2-) | beetle trap (only litter) | breeder farms | *Salmonella* enterica subsp. enterica serovar Senftenberg strain CVM 20749 | 98.61 | 94.64 |
| MACK32 | 18 | Kentucky | fly paper inside | breeder farms | *Salmonella* enterica subsp. enterica serovar Kentucky strain CVM 30177 | 93.35 | 99.93 |
| MACK33 | 23 | Kentucky | beetle trap (only litter) | breeder farms | *Salmonella* enterica subsp. enterica serovar Kentucky strain CVM 30177 | 93.37 | 99.92 |
| MACK34 | 47 | Kentucky | swab from nest box | breeder farms | *Salmonella* enterica subsp. enterica serovar Kentucky strain CVM 30177 | 93.37 | 99.92 |
| MACK35 | 51 | Kentucky | swab from egg collection conveyer belt | breeder farms | *Salmonella* enterica subsp. enterica serovar Kentucky strain CVM 30177 | 93.37 | 99.92 |
| MACK36 | 59 | Kentucky | bootswab | breeder farms | *Salmonella* enterica subsp. enterica serovar Kentucky strain CVM 30177 | 93.72 | 99.78 |
| MACK37 | 64 | Kentucky | fan exhaust swab inside | breeder farms | *Salmonella* enterica subsp. enterica serovar Kentucky strain CVM 30177 | 93.72 | 99.78 |
| MACK38 | 78 | Kentucky | boot swab | breeder farms | *Salmonella* enterica subsp. enterica serovar Kentucky strain CVM 30177 | 93.36 | 99.9 |
| MACK39 | 81 | Kentucky | swab from nest box | breeder farms | *Salmonella* enterica subsp. enterica serovar Kentucky strain CVM 30177 | 93.36 | 99.9 |
| MACK40 | 86 | Kentucky | dirty egg wipe paper | breeder farms | *Salmonella* enterica subsp. enterica serovar Kentucky strain CVM 30177 | 93.36 | 99.9 |
| MACK41 | 93 | Kentucky | fly paper outside | breeder farms | *Salmonella* enterica subsp. enterica serovar Kentucky str. SA20030505 | 97.08 | 99.87 |
| MACK42 | 114 | Enteritidis | water drainage | breeder farms | *Salmonella* enterica subsp. enterica serovar Enteritidis str. EC20090641 | 97.83 | 99.98 |
| MACK43 | 128 | Typhimurium | soil sample | breeder farms | *Salmonella* enterica subsp. enterica serovar Typhimurium strain SAP17-7699 | 97.37 | 99.31 |
| MACK44 | 138 | Typhimurium | water drainage | breeder farms | *Salmonella* enterica subsp. enterica serovar Typhimurium strain SAP17-7699 | 86.09 | 99.3 |
| MACK45 | 143 | Typhimurium | water puddles | breeder farms | *Salmonella* enterica subsp. enterica serovar Typhimurium strain SAP17-7699 | 97.36 | 99.31 |
| MACK46 | 17 | Kentucky | swab from egg collection conveyer belt | breeder farms | *Salmonella* enterica subsp. enterica serovar Kentucky strain CVM 30177 | 93.36 | 99.92 |
| MACK47 | 101 | Kentucky | crease in curtain, wildbird/rodent feces | breeder farms | *Salmonella* enterica subsp. enterica serovar Kentucky strain CVM 30177 | 95.65 | 99.93 |
| MACK48 | 102 | Kentucky | wildbird feces | breeder farms | *Salmonella* enterica subsp. enterica serovar Kentucky strain CVM 30177 | 93.33 | 99.93 |
| MACK49 | 54 | Kentucky | carcass rinses (pot-picking) | Processing plant | *Salmonella* enterica subsp. enterica serovar Kentucky str. SA20030505 | 97.08 | 99.87 |
| MACK50 | 55 | Kentucky | carcass rinses (pot-picking) | processing plant | *Salmonella* enterica subsp. enterica serovar Kentucky strain CVM 30177 | 95.68 | 99.92 |
| MACK51 | 59 | Kentucky | carcass rinses (pot-picking) | processing plant | *Salmonella* enterica subsp. enterica serovar Kentucky str. SA20030505 | 97.08 | 99.88 |
| MACK52 | 86 | Kentucky | carcass rinses (pot-picking) | processing plant | *Salmonella* enterica subsp. enterica serovar Kentucky str. SA20030505 | 97.1 | 99.69 |
| MACK53 | 106 | Kentucky | carcass rinses (pot-chilling) | processing plant | *Salmonella* enterica subsp. enterica serovar Kentucky str. SA20030505 | 97.17 | 99.87 |
| MACK54 | 107 | Kentucky | carcass rinses (pot-chilling) | processing plant | *Salmonella* enterica subsp. enterica serovar Kentucky str. SA20030505 | 97.08 | 99.87 |
| MACK55 | 207 | Kentucky | carcass rinses (pot-chilling) | processing plant | *Salmonella* enterica subsp. enterica serovar Kentucky str. SA20030505 | 97.08 | 99.87 |
| MACK56 | 115 | Kentucky | swab from moving belt (live birds hang area) | processing plant | *Salmonella* enterica subsp. enterica serovar Kentucky str. SA20030505 | 97.07 | 99.87 |
| MACK57 | 116 | Uganda | swab from moving belt (live birds hang area) | processing plant | *Salmonella* enterica strain CFSAN060807 | 97.83 | 98.15 |
| MACK58 | 119 | Alachua or IIIa 35:z4, z23:- | swab from moving belt (live birds hang area) | processing plant | *Salmonella* enterica subsp. enterica serovar Worthington strain CVM 30148 | 79.63 | 80.22 |
| MACK59 | 120 | Kentucky | fresh feces from live birds hang area | processing plant | *Salmonella* enterica subsp. enterica serovar Kentucky str. SA20030505 | 97.07 | 99.87 |
| MACK60 | 130 | Kentucky | swab from moving belt (loading dock area) | processing plant | *Salmonella* enterica subsp. enterica serovar Kentucky strain CVM 30177 | 93.37 | 99.92 |
| MACK61 | 131 | Kentucky | swab from moving belt (loading dock area) | processing plant | *Salmonella* enterica subsp. enterica serovar Kentucky str. SA20030505 | 97.09 | 99.87 |
| MACK62 | 132 | Kentucky | swab from moving belt (loading dock area) | processing plant | *Salmonella* enterica subsp. enterica serovar Kentucky str. SA20030505 | 97.09 | 99.87 |
| MACK63 | 149 | Alachua or IIIa 35:z4, z23:- | transport cage swab just before loading birds | transport | *Salmonella* enterica subsp. enterica serovar Worthington strain CVM 30148 | 80.43 | 80.17 |
| MACK64 | 160 | Kentucky | transport cage while in trucks | transport | *Salmonella* enterica subsp. enterica serovar Kentucky strain CVM 30177 | 93.34 | 99.92 |
| MACK65 | 165 | Kentucky | transport cage while in trucks | transport | *Salmonella* enterica subsp. enterica serovar Kentucky str. SA20030505 | 97.08 | 99.87 |
| MACK66 | 166 | Kentucky | transport cage while in trucks | transport | *Salmonella* enterica subsp. enterica serovar Kentucky str. SA20030505 | 97.08 | 99.87 |
| MACK67 | 220 | Kentucky | carcass rinses (post-picking) | processing plant | *Salmonella* enterica subsp. enterica serovar Kentucky strain CVM 30177 | 93.37 | 99.92 |
| MACK68 | 221 | Kentucky | carcass rinses (post-picking) | processing plant | *Salmonella* enterica subsp. enterica serovar Kentucky strain CVM 30177 | 93.37 | 99.92 |
| MACK69 | 222 | Kentucky | carcass rinses (post-picking) | processing plant | *Salmonella* enterica subsp. enterica serovar Kentucky strain CVM 30177 | 93.32 | 99.93 |
| MACK70 | 276 | Kentucky | carcass rinses (post-chilling) | processing plant | *Salmonella* enterica subsp. enterica serovar Kentucky strain CVM 30177 | 93.37 | 99.92 |
| MACK71 | 283 | Kentucky | carcass rinses (post-chilling) | processing plant | *Salmonella* enterica subsp. enterica serovar Kentucky strain CVM 30177 | 95.66 | 99.91 |
| MACK72 | 330 | Kentucky | swab from moving belt (live birds hang area) | processing plant | *Salmonella* enterica subsp. enterica serovar Kentucky strain CVM 30177 | 93.37 | 99.92 |
| MACK73 | 331 | Enteritidis | swab from moving belt (live birds hang area) | processing plant | *Salmonella* enterica subsp. enterica serovar Enteritidis str. EC20090641 | 97.83 | 99.98 |
| MACK74 | 335 | Kentucky | swab from moving belt (live birds hang area) | processing plant | *Salmonella* enterica subsp. enterica serovar Kentucky strain CVM 30177 | 93.32 | 99.93 |
| MACK75 | 340 | Alachua or IIIa 35:z4, z23:- | fresh feces from live birds hang area | processing plant | *Salmonella* enterica subsp. enterica serovar Worthington strain CVM 30148 | 79.64 | 80.22 |
| MACK76 | 350 | Alachua or IIIa 35:z4, z23:- | swab from floor of transport cages | transport | *Salmonella* enterica subsp. enterica serovar Worthington strain CVM 30148 | 79.66 | 80.22 |
| MACK77 | 354A | Alachua or IIIa 35:z4, z23:- | swab from floor of transport cages | transport | *Salmonella* enterica subsp. enterica serovar Worthington strain CVM 30148 | 79.63 | 80.22 |
| MACK78 | 354C | Alachua or IIIa 35:z4, z23:- | swab from floor of transport cages | transport | *Salmonella* enterica subsp. enterica serovar Worthington strain CVM 30148 | 79.64 | 80.22 |
| MACK79 | 356 | Kentucky | swab from floor of transport cages | transport | *Salmonella* enterica subsp. enterica serovar Kentucky str. SA20030505 | 98.73 | 99.87 |
| MACK80 | 4C | Kentucky | swab from cage floor on truck | transport | *Salmonella* enterica subsp. enterica serovar Kentucky strain CVM 30177 | 95.66 | 99.92 |
| MACK81 | 7A | Kentucky | swab from cage floor on truck | transport | *Salmonella* enterica subsp. enterica serovar Kentucky strain CVM 30177 | 95.66 | 99.92 |
| MACK82 | 9A | Kentucky | swab from cage floor on truck | transport | *Salmonella* enterica subsp. enterica serovar Kentucky strain CVM 30177 | 95.66 | 99.91 |
| MACK83 | 29 | Kentucky | fecal sample from truck | transport | *Salmonella* enterica subsp. enterica serovar Kentucky strain CVM 30177 | 95.65 | 99.91 |
| MACK84 | 31 | Kentucky | fecal sample from truck | transport | *Salmonella* enterica subsp. enterica serovar Kentucky strain CVM 30177 | 93.37 | 99.91 |
| MACK85 | 37 | Kentucky | fecal sample from truck | transport | *Salmonella* enterica subsp. enterica serovar Kentucky str. SA20030505 | 96.93 | 99.53 |
| MACK86 | 56 | Kentucky | fecal sample from live birds hang area | processing plant | *Salmonella* enterica subsp. enterica serovar Kentucky strain CVM 30177 | 93.32 | 99.92 |
| MACK87 | 62 | Kentucky | swab from moving belt (live birds hang area) | processing plant | *Salmonella* enterica subsp. enterica serovar Kentucky str. SA20030505 | 97.06 | 99.69 |
| MACK88 | 63 | Kentucky | swab from moving belt (live birds hang area) | processing plant | *Salmonella* enterica subsp. enterica serovar Kentucky strain CVM 30177 | 95.66 | 99.91 |
| MACK89 | 64 | Uganda | swab from moving belt (live birds hang area) | processing plant | *Salmonella* enterica strain CFSAN060807 | 97.82 | 98.14 |
| MACK90 | 75 | Kentucky | carcass rinses (post-picking) | processing plant | *Salmonella* enterica subsp. enterica serovar Kentucky str. SA20030505 | 97.07 | 99.87 |
| MACK91 | 79 | Kentucky | carcass rinses (post-picking) | processing plant | *Salmonella* enterica subsp. enterica serovar Kentucky str. SA20030505 | 97.08 | 99.88 |
| MACK92 | 80 | Kentucky | carcass rinses (post-picking) | processing plant | *Salmonella* enterica subsp. enterica serovar Kentucky str. SA20030505 | 97.07 | 99.87 |
| MACK94 | 253 | Kentucky | fan exhaust swab inside | broiler farms | *Salmonella* enterica subsp. enterica serovar Kentucky str. SA20030505 | 97.09 | 99.87 |
| MACK95 | 254 | Kentucky | fan exhaust swab inside | broiler farms | *Salmonella* enterica subsp. enterica serovar Kentucky strain CVM 30177 | 93.36 | 99.9 |
| MACK96 | 529 | Enteritidis | fan exhaust swab inside | broiler farms | *Salmonella* enterica subsp. enterica serovar Enteritidis str. EC20090641 | 97.83 | 99.98 |
| MACK97 | 110 | Kentucky |  |  | *Salmonella* enterica subsp. enterica serovar Kentucky strain CVM 30177 | 93.37 | 99.92 |
| MACK105 | 14 | Enteritidis | ants | broiler farms | *Salmonella* enterica subsp. enterica serovar Enteritidis str. EC20120005 | 91.01 | 99.98 |
| MACK106 | 1109 | Montevideo | drainage water | broiler farms | *Salmonella* enterica subsp. enterica serovar Montevideo str. CDC 2011K-1674 | 98.08 | 99.15 |
| MACK107 | 1110 | Montevideo | drainage water | broiler farms | *Salmonella* enterica subsp. enterica serovar Montevideo str. CDC 2011K-1674 | 99.62 | 99.14 |
| MACK108 | 1111 | Montevideo | feces, deer | broiler farms | *Salmonella* enterica subsp. enterica serovar Montevideo str. CDC 2011K-1674 | 98.8 | 99.15 |
